# Bombesin Analogue-Mediated Delivery Preferentially Enhances the Cytotoxicity of a Mitochondria-Disrupting Peptide in Tumor Cells

**DOI:** 10.1371/journal.pone.0057358

**Published:** 2013-02-25

**Authors:** Hao Yang, Huawei Cai, Lin Wan, Shan Liu, Shengfu Li, Jingqiu Cheng, Xiaofeng Lu

**Affiliations:** Key Lab of Transplant Engineering and Immunology, Ministry of Health, West China Hospital, Sichuan University, Chengdu, China; University of Pittsburgh, United States of America

## Abstract

Tumor-homing peptides that recognize specific markers on tumor cells have shown potential as drug carriers for targeted cancer therapy. Bombesin receptors are frequently overexpressed or ectopically expressed in a wide range of human tumors. Bombesin and its analogues have been widely used as drug carriers for tumor imaging and tumor therapy. However, the cargos used in previous studies, including radioactive and chemotherapeutic agents, are usually small molecules. Mitochondrial-disrupting peptides depolarize the mitochondria and trigger apoptosis after entering tumor cells. We are interested in whether the bombesin analogue, Bn(6–14), which contains a bombesin receptor-binding motif, can specifically deliver the mitochondria-disrupting peptide, B28, to tumor cells. To this end, we created a chimeric peptide, B28Bn(6–14), by conjugating B28 to Bn(6–14) at its N-terminus. The cytotoxicity of B28Bn(6–14) in tumor cells was much stronger than unconjugated B28. The IC_50_ values of B28Bn(6–14) in tumor cells (1.7–3.5 µM) were approximately 10 times lower than B28. However, conjugation of B28 to Bn(2–7), which lacks the bombesin receptor-binding motif, did not increase its cytotoxicity. In addition, the IC_50_ values of B28Bn(6–14) in tumor cells (1.7–3.5 µM) was 3–10 times lower than in normal cells (10.8–16.8 µM). We found that selective binding of B28Bn(6–14) to tumor cells is Bn(6–14)-dependent. Upon entering the tumor cell, B28Bn(6–14) accumulated in the mitochondria and triggered caspase-dependent apoptosis. Intratumoral and intraperitoneal administration of B28Bn(6–14) substantially suppressed the growth of DU145 tumor xenografts in mice. These results demonstrate that Bn(6–14) is able to deliver the mitochondria-disrupting peptide to tumor cells, and B28Bn(6–14) should be further developed as novel anti-cancer agent.

## Introduction

Traditional chemotherapy usually has very limited selectivity toward tumor tissues and frequently induces the emergence of multiple drug resistance due to the requirement for high drug doses [1]. Developing strategies to exhibit selective toxicity toward tumor cells relative to normal cells is currently one of the major challenges in anticancer therapy. Targeted delivery of anticancer agents to malignant cells based on tumor biomarkers has the potential to increase therapeutic efficacy while decreasing dose-limiting side effects [2], [3]. Tumor-homing peptide ligands represent a promising approach for the specific delivery of diagnostic and therapeutic agents, as the ligands show a strong affinity toward biomarker receptors overexpressed on tumor cells or tumor vasculature [4], [5].

One strategy for targeted drug delivery by using tumor-homing peptides is the coadministration of drugs and the peptides as separate entities without conjugation. After the tumor-homing peptide selectively accumulates in tumor tissues, an additional motif in the peptide, such as CendR, induces leakage of the tumor vasculature by affecting the integrity of angiogenic endothelial cells and triggers the targeted delivery of the bystander drugs into tumor tissues [6], [7]. On the other hand, most tumor-homing peptides, as leader moieties, can be conjugated to diverse cargos, including cytotoxic drugs, imaging agents, and various nanoparticles, for tumor diagnosis and targeted treatment. Based on conjugation, many tumor-homing peptide-directed agents have been used in the clinic or are undergoing clinical trials [4], [5], [8], [9]. For instance, radiolabeled somatostatin analogues are currently used for cancer imaging and therapy. Among these analogues, ^111^In-penetreotide based somatostatin receptor scintigraphy is a standard clinical procedure to determine the localization of neuroendocrine tumors [9], [10]. However, the overexpression of somatostatin receptors is limited to neuroendocrine tumors [11].

Bombesin, which is an amidated tetradecapeptide isolated from frog skin, is another attractive vehicle for tumor-targeting delivery. Bombesin shares the same, or a similar, seven C-terminal amino acid sequence with gastrin-releasing peptide and neuromedin B, respectively. Therefore, the bombesin receptor family in mammals is comprised of gastrin-releasing peptide receptor (GRPR), neuromedin B receptor (NMBR), and bombesin receptor subtype 3 (BRS-3) [12]. These bombesin receptors, especially GRPR, are frequently overexpressed or ectopically expressed in many common malignancies, including lung cancer, prostate cancer, breast cancer, pancreatic cancer, head/neck cancer, colon cancer, uterine cancer, ovarian cancer, renal cell cancers, glioblastomas, neuroblastomas, gastrointestinal carcinoids, intestinal carcinoids, and bronchial carcinoids. Thus, there is special interest in developing bombesin receptor-mediated agents to treat these tumors [8], [12]. Currently, numerous radiolabeled bombesin analogues are undergoing investigation for tumor imaging and radiotherapy. Some ^99m^Tc or ^68^Ga-labeled analogues were tested in healthy volunteers or patients for diagnostic purposes [8]. In addition, a few nonradiolabeled analogues that were constructed by conjugating bombesin analogues to chemotherapeutic agents, such as camptothecin, doxorubicin, and paclitaxel, have successfully increased the selectivity or efficacy of these drugs in preclinical studies [13], [14], [15].

Previous studies demonstrated that peptide fragments containing residues 7–9 in the C terminus of bombesin show high affinity toward bombesin receptors [16]. These bombesin analogues have been widely studied as vehicles of tumor-imaging and targeted therapy agents. However, the cargos that have been used in these studies primarily include small molecule radiolabeled and chemotherapeutic agents [8], [17], [18], [19]. Limited biomolecules, such as marine toxin, diphtheria toxin and nanoparticles loading siRNA, have also been fused to bombesin analogues for targeted delivery [8]. Mitochondria are considered to be the powerhouse of the cells and one of the crucial signal regulators for cell survival and death [20]. Mitochondria-disrupting peptides can efficiently activate mitochondrial membrane permeabilization (MMP) and disruption, and trigger apoptosis after being delivered into tumor cells by drug carriers, such as tumor cell-selective peptides or antibodies [21], [22]. Because C-terminal fragments of bombesin containing the receptor-binding motif have been used as vehicles for small molecules, we sought to determine whether these bombesin analogues could be used for targeted delivery of a mitochondria-disrupting peptide.

In this study, we selected the mitochondria-disrupting peptide, truncated BMAP-28 (B28) [23], as a cargo and constructed the chimeric peptide by coupling B28 to a bombesin analogue, i.e., Bn(6–14) containing the bombesin receptor-binding motif. We found that Bn(6–14) significantly enhanced the selective cytotoxicity of B28 for tumor cells *in vitro* and *in vivo*. The Bn(6–14)-directed chimeric peptide was localized at the mitochondria once internalized into tumor cells, and it induced caspase-dependent apoptosis in tumor cells.

## Materials and Methods

### Reagents

The LIVE/DEAD BacLight Bacterial Viability Kit, MitoTracker® Red CMXRos, FITC-Annexin V/Propidium Iodide (PI) Kit, and 5,5′,6,6′-tetrachloro-1,1′,3,3′-tetraethyl-benzamidazolocarbocyanin iodide dye (JC-1) were purchased from Invitrogen, USA. Other reagents used in this study included fetal bovine serum (FBS, GIBCO-BRL, USA), the Terminal Nucleotidyl Transferase–mediated Nick End Labeling (TUNEL) staining kit (Roche, USA), Cytotox 96 non-radioactive cytotoxicity assay kit (Promega, USA), Pan-caspase inhibitor Z-VAD-FMK (R&D Systems, USA), Alexa Fluor 488-labeled polyclonal antibody against human cleaved caspase-3 (Cell Signaling, USA), and Cell Counting Kit-8 (CCK-8) (Dojindo Chemical, Japan).

### Cell Culture

Human prostate cancer cells (DU145 and PC-3), human breast cancer cells (MCF-7), human skin fibroblast cells (HSF), human lung fibroblast cells (MRC-5), and human prostate stromal cells (PrSC) were from the American Type Culture Collection. Human prostatic smooth muscle cells (SMC) and human breast cancer cells (MDA-MB-435S) were obtained from the Cell Bank of the Chinese Academy of Science. All of the cells were grown in RPMI 1640 supplemented with 10% FBS, 2 mmol/L L-glutamine, 100 U/mL penicillin, and 100 µg/mL streptomycin at 37°C with 5% CO_2_.

### Design and Synthesis of Peptide Conjugates

It is known that bombesin analogues containing residues 7–9 of the C-terminal bombesin show high affinity for the bombesin receptor. Of these analogs, Bn(6–14), which contains 9 residues of bombesin C terminus, was the most frequently used vehicle. The truncated BMAP28, (i.e., B28) has the ability to disrupt mitochondria. To investigate the ability of Bn(6–14) to target the delivery of B28 to tumor cells, the chimeric peptide, B28Bn(6–14), was constructed by conjugating B28 to Bn(6–14) at the N terminus. Simultaneously, B28 was also conjugated to another bombesin-derived peptide, Bn(2–7), which lacks the receptor-binding motif, to construct chimeric peptide, B28Bn(2–7). Other peptides, including unconjugated Bn(6–14), Bn(2–7), and B28 were also used in this study (Table 1). Before these peptides were synthesized, several online methods including MLRC (multivariate linear regression combination), PHD (neural network system), and SOPM (self-optimized prediction method) were utilized to predict the secondary structure of the peptide, according to the description by Combet et al. [24], and the application by Do et al. [25]. The prediction was performed on the Pole Bio-Informatique Lyonnais-Network Protein Sequence Analysis Web Server (http://npsa-pbil.ibcp.fr) following the step by step online guide. All peptides were custom synthesized by Genescript Inc., (Nanjing, China) using standard solid-phase fluorenylmethoxycarbonyl (Fmoc) chemistry techniques. The purity of the peptides (>95%) was analyzed by reversed-phase high performance liquid chromatography, and their masses were assayed by matrix-assisted laser desorption ionization-time of flight mass spectrometry. Fluorescein isothiocyanate (FITC) labels were linked to the N-terminus of the peptide by introducing 5-carbonxyfluorescein during the final synthetic cycle. All peptides were dissolved in PBS (137 mM NaCl, 2.68 mM KCl, 8.09 mM Na_2_HPO_4_, 1.76 mM KH_2_PO_4_, pH 7.4) or RPMI 1640 and stored at −80°C.

**Table 1:** Amino acid (aa) sequences of the peptides used in this study.

Peptide	Sequence	Length(aa)	Molecular Weight(Da)
Bn(6–14)	NQWAVGHLM-Amide	9	1054
Bn(2–7)	QRLGNQ-Amide	6	714
B28	GGLRSLGRKILRAWKKYG	18	2059
B28Bn(6–14)	GGLRSLGRKILRAWKKYGNQWAVGHLM-Amide	27	3095
B28Bn(2–7)	GGLRSLGRKILRAWKKYGQRLGNQ-Amide	24	2755

### Circular Dichroism Analysis of Peptide Secondary Structure

Each peptide, at the concentration of 0.5 mg/ml, was dissolved in 2 mM phosphate buffer, pH 7.4. The circular dichroism (CD) spectra of the peptides were recorded from 190 nm to 400 nm on a Model 400 Circular Dichroism Spectrophotometer (Aviv Biomedical, Inc.) at 25°C. Background scans were collected in buffer alone and subtracted from the peptide scan. Each sample was performed in triplicate, and the CD spectrum was obtained with the calculated average.

### Cytotoxicity Assay

Cells were seeded at a density of 1×10^4^ per well in a 96-well plate, were grown overnight and were co-incubated at 37°C for 1 h with increasing concentrations of the peptide in 100 µl RPMI 1640 supplemented with 2% FBS. Subsequently, the peptide solution was replaced by 100 µl fresh medium. To determine cell viability, 10 µl CCK-8 was added to the well, and the cultures were incubated for an additional 2–4 h. The absorbance was then measured at 450 nm (with a reference wavelength at 620 nm), and cell viability was calculated as the percentage of the value relative to the absorbance of the cells incubated without peptide. The IC_50_ values of the peptides were calculated from the respective cell viability curves. Simultaneously, the LIVE/DEAD BacLight bacterial viability kit was used to visualize the cytotoxicity of the peptides. Combined incubation with SYTO 9 (15 µM) and propidium iodide (PI, 2.5 µg/ml) stained the living cells fluorescent green and the dead cells fluorescent red due to their distinct cell membrane permeability.

### Peptide Cellular Uptake and Localization in Mitochondria

To quantitate the cellular uptake of the peptide by fluorescence-activated cell sorting (FACS), cells were detached with a non-enzymatic cell dissociation solution (Sigma Aldrich), washed with culture medium and suspended in PBS containing 0.5% FBS. Subsequently, 1.5×10^5^ cells were treated with 300 µl of 5 µM FITC-labeled peptides at 37°C for 30 min. The cells were collected by centrifugation, washed twice with PBS containing 0.5% FBS and subjected to FACS analysis. An unrelated FITC-labeled goat anti-rabbit IgG was used as a negative control. To further examine the cellular localization of the peptide to the mitochondria, cells were plated at a density of 3×10^4^ cells/cm^2^ and incubated with 5 µM FITC-labeled peptides in PBS containing 0.5% FBS at 37°C for 15–30 min. The mitochondrion-selective fluorescent probe, Mitotracker CMXRos (10 ng/ml), was added for the last 15 min of the incubation. After quenching the cell surface-bound FITC peptide with 200 µg/ml trypan blue, as described previously [21], the cells were gently washed with PBS containing 0.5% FBS and observed under a fluorescence microscope.

### Mitochondrial Depolarization Assessment

Cells were detached, washed with medium, and 1.5×10^5^ cells were suspended in 300 µl of RMPI 1640 supplemented with 10% FBS. Then, the cells were treated with the peptide for the indicated period of time and stained with 2 µM JC-1 dye for 15 min. After being washed twice with PBS, the cells were analyzed by FACS to evaluate the change in mitochondrial transmembrane potential. JC-1 forms red fluorescent J-aggregates upon localization in healthy mitochondria, whereas the dispersed monomeric form of the dye in the cytoplasm fluoresces green [26]. Hence, the loss of mitochondrial transmembrane potential results in a decrease in the ratio of red to green fluorescence. To visualize the mitochondria under a fluorescence microscope, attached cells, at a density of 3×10^4^ cells/cm^2^, were treated with the peptide over a time course. Subsequently, JC-1 and DAPI were used to identify the mitochondria and nuclei, respectively.

### Detection of Apoptosis and Necrosis

Phosphatidylserine exposed on the outer membrane reflects the early stages of apoptosis and can be detected by FITC-Annexin V and PI staining. The cells were detached and washed with medium. One-hundred and fifty thousand cells were suspended in 300 µl of RMPI 1640 supplemented with 10% FBS and treated with the peptide at the indicated concentration and time points. Then, the cells were subjected to dual staining with FITC-Annexin V and PI, according to the manual. The cells were observed under a fluorescence microscope and subjected to FACS analysis. Cells showing Annexin V−/PI-, Annexin V+/PI-, and Annexin V+/PI+ signals are considered to be living, early stage apoptotic, and either late stage apoptotic or necrotic, respectively. To assess DNA damage during apoptosis, the TUNEL assay was performed in accordance with the manufacturer’s instruction. Briefly, after treatment for 30 min-1 h with the peptide, cells were washed twice with PBS, fixed with 4% paraformaldehyde for 10–15 min at room temperature, permeabilized with 0.1% Triton X-100 for 2 min on ice, incubated in the reaction mixture and observed under a fluorescence microscope. FITC labeled nucleotides incorporated into the nuclei reflects DNA degradation. For investigating the activation of caspases, cells were preincubated with the Pan-caspase inhibitor, Z-VAD-FMK (100 µM), for 2 h before peptide treatment in the cytotoxicity assay. Immunofluorescence staining of caspase-3 was performed by using the Alexa Fluor 488-conjugated antibody against human cleaved caspase-3. The activation of caspase-3 was further verified by using a caspase-3 colorimetric assay kit (Genescript, Nanjing, China) with TNF-related apoptosis-inducing ligand (TRAIL) as a positive control. The activity of caspase-3 was presented as fold of peptide-treated cells vs untreated cells.

Necrosis was evaluated with the Cytotox 96 non-radioactive cytotoxicity assay kit by detecting the release of lactate dehydrogenase (LDH) from necrotic cells. Ten-thousand cells were plated in a 96-well plate and treated with the peptide in 100 µl of RMPI 1640 supplemented with 2% FBS for 10 min-2 h, followed by LDH detection with 50 µl of culture supernatant from the treated cells, according to the manufacturer’s instruction. The LDH level was presented as the percentage relative to that of cells treated with 0.9% Triton X-100.

### Peptide-induced Hemolysis Analysis

Hemolysis was assessed according to a procedure described previously, with some modifications. Human erythrocytes from healthy donors were isolated by low-density (Ficoll-Paque PLUS, 1.077 g/mL, GE Healthcare) gradient centrifugation, washed three times with PBS and suspended in PBS at the concentration of 4% (volume ratio). In triplicate, erythrocyte suspensions were treated with the peptide in a 96-well plate at 37°C from 30 min to 16 h, and the absorbance was measured at 540 nm [27]. Hemolysis was presented as the percentage of the value relative to the absorbance of erythrocytes treated with 0.1% Triton X-100.

### *In Vivo* Tumor Xenograft Models

All of the protocols used for in vivo experiments were approved by the University Animal Care and Use Committee. Four to six-week-old male BALB/c nu/nu mice were purchased from the University Animal Production Center. Seven million DU145 cells suspended in 200 µl PBS were subcutaneously injected into the left flank region of mice. On day 7–9 post inoculation, the mice were randomized into three groups (n = 5) and intraperitoneally (i.p.) received 15 mg/kg B28Bn(6–14), B28 or an equivalent volume of PBS once daily for 7 consecutive days. Alternatively, the mice intratumorally received 5 mg/kg drug once daily for 5 consecutive days. The tumor volume was calculated as length×width^2^×0.5. At the end of i.p. therapy, the animals were sacrificed and the major organs were excised, paraffin-embedded, sectioned, and stained with hematoxylin/eosin (H&E) for the examination of histopathologic architecture. To evaluate the cytotoxicity on tumor cells *in vivo*, a single dose of 50 µg of peptide in 50 µl of PBS was injected into the tumor graft (200–300 mm^3^), according to the method described previously [21]. At 24 h post-injection, the animals were sacrificed, and the tumor tissues were routinely stained with H&E.

### Statistical Analysis

Each experimental condition was performed in triplicate or repeated at least three times. The data are presented as the mean±SD. Student’s *t* test was used to evaluate differences in the cell viability in caspase inhibition assays. One-way ANOVA was applied to compare differences in tumor growth *in vivo*. Significant differences were demonstrated to exist at *P*<0.05.

## Results

### Design and Structural Analysis of Cytotoxic Peptide Conjugates

**Figure 1:**
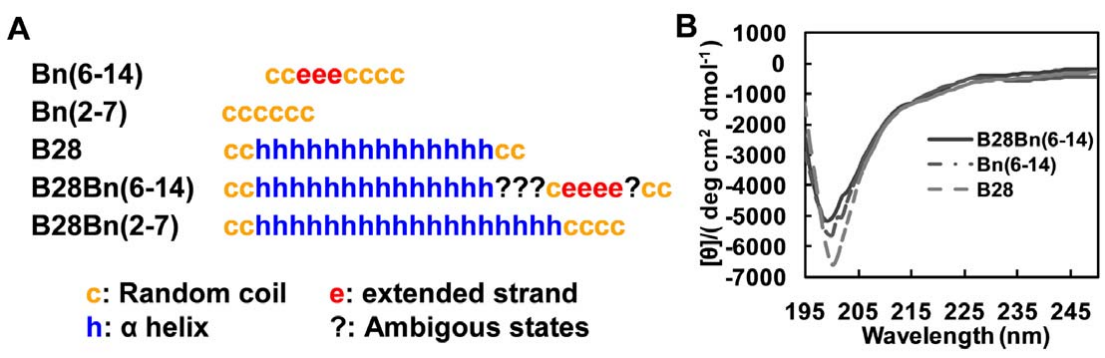
Peptide secondary structure prediction and measurement. **A**. A peptide secondary structure consensus prediction was performed based on the amino acid sequence using a set of online methods, including MLRC, PHD, and SOPM. Each random coil (c), extended strand (e), α helix (h), and ambiguous state (?) corresponded to an amino acid in the peptide sequence. **B**. The actual secondary structure contents of the peptides were measured by far-ultraviolet (UV) circular dichroism (CD) spectrum. The CD spectra of B28 (dashed line), Bn(6–14) (dot-dashed line) and B28Bn(6–14) (solid line) were recorded in 2 mM sodium phosphate buffer (pH 7.4) at 25°C.

For targeted delivery of the cytotoxic peptide to tumor cells using the bombesin analogue, Bn(6–14), B28Bn(6–14) was constructed by conjugating the mitochondria-disrupting peptide, B28, at the N terminus of Bn(6–14). B28Bn(2–7) was also constructed by linking B28 to Bn(2–7), which lacks the receptor-binding motif (Table 1). As presented in figure 1A, the secondary structure consensus prediction showed that unconjugated Bn(6–14) and Bn(2–7) comprise random coil and extended strand structures. Unconjugated B28 is composed of a long α helix at a proportion of 78%. Once conjugated, Bn(6–14) and B28 virtually maintained their respective secondary structures in B28Bn(6–14). Conjugation of B28 to Bn(2–7) forms an longer α helix in B28Bn(2–7) than that in B28 and B28Bn(6–14). The CD spectra of B28, Bn(6–14) and B28Bn(6–14) in phosphate buffer reflected the actual structure of these peptides. These data displayed a similar single minimum, around 200 nm for each peptide, which is characteristic of a random arrangement (Figure 1B). The structural prediction and measurement suggest that both B28 and Bn(6–14) in B28Bn(6–14) show homologous secondary structure versus their unconjugated forms.

### Bn(6–14) Greatly Enhances the Cytotoxicity of B28 in Tumor Cells

**Figure 2:**
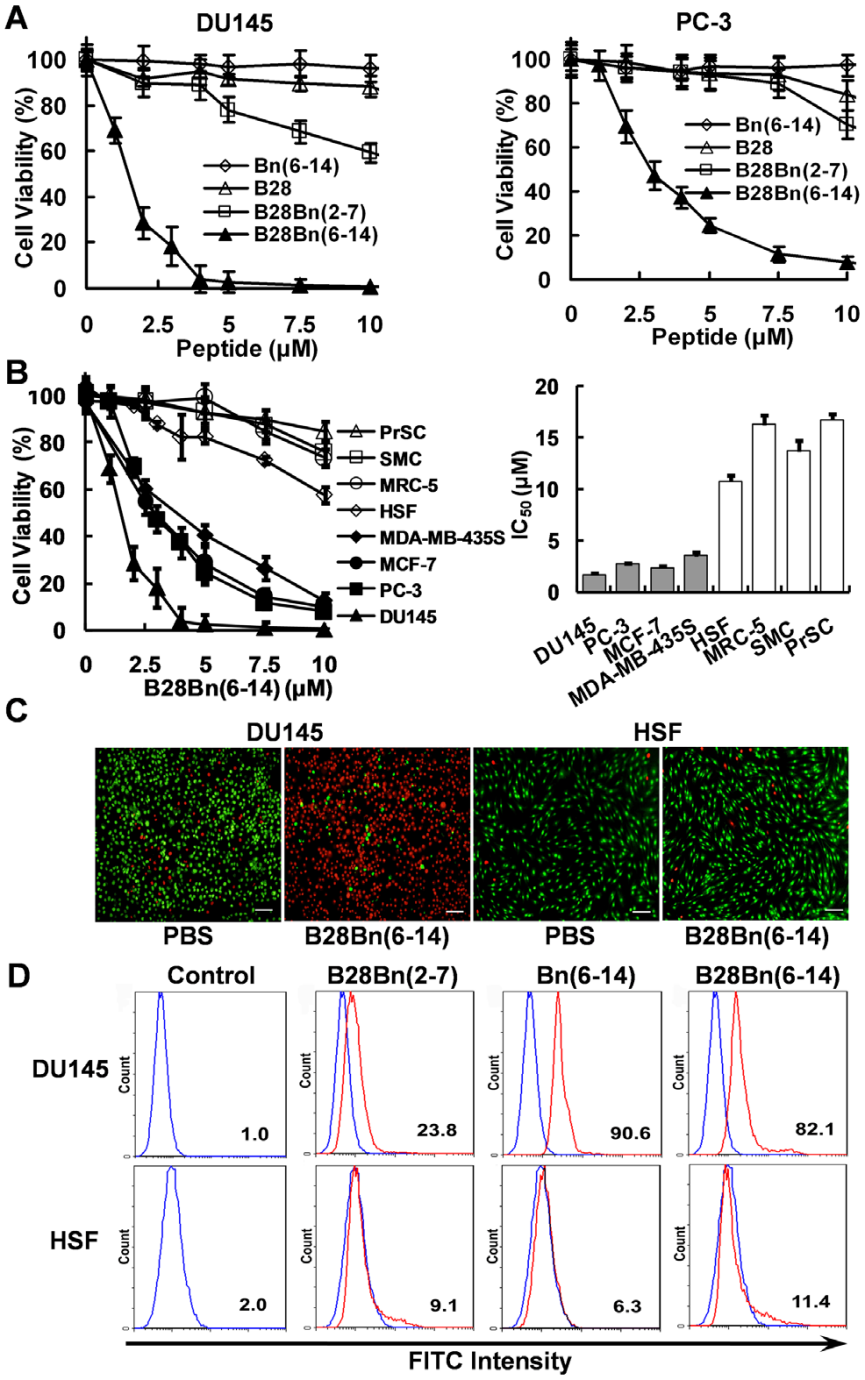
Targeted cytotoxicity of B28Bn(6–14) on tumor cells is directed by the Bn(6–14) motif. A . Comparison of the cytotoxicity of Bn(6–14), B28, the conjugate B28Bn(6–14) and B28Bn(2–7). DU145 and PC-3 cells were treated with increasing concentrations of the peptide for 1 h and cell viability was measured. **B**. Comparison of the cytotoxicity of B28Bn(6–14) in tumor and non-malignant cells (**left panel**). The IC_50_ values were calculated from the respective cell viability curves (**right panel**). **C**. Live/Dead assay for DU145 tumor cells and HSF normal cells. Cells were dual stained with SYTO 9 and PI after treatment with 5 µM B28Bn(6–14) for 45 min. The living cells fluoresced green, while the dead cells fluoresced red. Scale bar is 50 µm. **D**. Cellular uptake of FITC-labeled B28Bn(2–7), Bn(6–14), and B28Bn(6–14) in DU145 and HSF cells were tested with FITC-labeled IgG1 as control. Cells were incubated with 5 µM FITC-labeled peptides (red line) or IgG1 (blue line) for 30 min, washed with PBS, and analyzed by FACS. The percentage of positive cells was indicated.

To examine the cytotoxicity of the Bn(6–14)-directed cytotoxic peptide, we chose human prostate cancer DU145 and PC-3 cells that overexpress bombesin receptors [28], [29]. Unconjugated B28 showed weak cytotoxicity (10∼20%) on DU145 and PC-3 at a high concentration (10 µM). Bn(6–14) exhibited no obvious cytotoxicity in either cell line (Figure 2 A). In contrast, the chimeric peptide, B28Bn(6–14), containing B28 and Bn(6–14), dramatically decreased cell viability (50%-90%) in DU145 and PC-3 cells at concentrations ranging from 2.5–5 µM. However, the chimeric peptide B28Bn(2–7), containing B28 and Bn(2–7), which lacks the receptor-binding motif, showed moderate cytotoxicity toward DU145 and PC-3. The loss of cell viability only reached 10%-20% after treatment with 5 µM B28Bn(2–7) and was less than 40% at 10 µM (Figure 2A). These results suggest that only the C-terminal bombesin sequence containing the bombesin receptor-binding motif, rather than its N-terminal sequence, greatly enhances the cytotoxicity of B28 in tumor cells. This finding implies that full length of bombesin is not necessary to deliver the cytotoxic peptide to tumor cells.

### B28Bn(6–14) Selectively Induces Tumor Cell Death by Enhancing Bn(6–14)-directed Preferential Binding to Tumor Cells

We next tested the cytotoxicity of B28Bn(6–14) on tumor and normal cells. Treatment of PC-3, DU145, MC-7, and MDA-MB-435S with increasing concentrations of B28Bn(6–14) resulted in significantly decreased cell viability. As shown in Figure 2B, the loss of cell viability reached 60–90% after treatment with 5 µM B28Bn(6–14) and over 90% when the concentration was increased to 10 µM (left panel). The mean IC_50_ values of B28Bn(6–14) for each tumor cell line varied from 1.7–3.5 µM (right panel). In contrast, normal cells, including HSF, PrSC, SMC, and MRC-5, were relatively resistant to B28Bn(6–14). Less than 20% of the normal cells died following treatment with 5 µM B28Bn(6–14). The mean IC_50_ values of B28Bn(6–14) for the normal cells were ranged from 10.8-16.8 µM. These results suggest that B28Bn(6–14) is selectively cytotoxic to tumor cells. The preferential cytotoxicity of B28Bn(6–14) toward tumor cells was also confirmed by live/dead staining with SYTO 9 and PI. DU145 tumor cells treated with 5 µM B28Bn(6–14) showed over 90% dead cells (red fluorescence), whereas HSF normal cells exhibited less than 10% cell death after treatment with the equivalent concentration of B28Bn(6–14) (Figure 2C). To investigate the mechanism for this selective cytotoxicity, we further detected the cellular uptake of the peptides in tumor and normal cells by FACS analysis. The B28-Bn(6–14) conjugate, B28Bn(6–14), bound to over 80% of the DU145 tumor cells at 5 µM but bound to approximately 10% of the HSF normal cells at the same concentration. Similarly, the unconjugated Bn(6–14) bound to over 90% of DU145 tumor cells compared to 6.3% of the HSF normal cells. In contrast, the control conjugate, B28Bn(2–7), in which B28 was conjugated to the N-terminal bombesin sequence, bound to a low proportion (10–20%) of both tumor and normal cells at the same concentration (Figure 2D). These data indicate that the Bn(6–14), rather than B28 motif, in B28Bn(6–14) contributed to the selective cellular uptake and subsequent preferential cytotoxicity of B28Bn(6–14) in tumor cells.

### B28Bn(6–14) Localizes to Mitochondria and Induces Mitochondrial Depolarization

**Figure 3:**
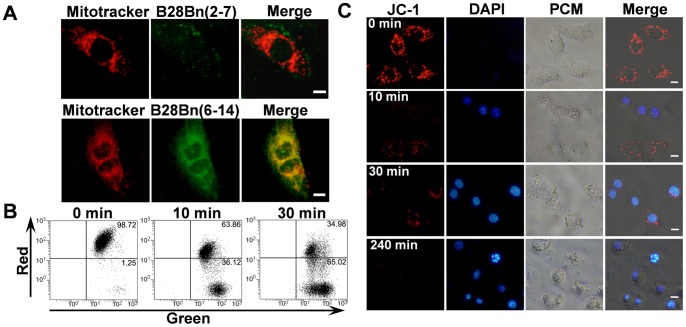
B28Bn(6–14)-induced loss of mitochondrial transmembrane potential. A . The mitochondrial localization of B28Bn(6–14) was assessed by co-localization of Mitotracker in DU145 cells. Cells were treated with 5 µM FITC-labeled B28Bn(6–14) for 15–30 min, and the mitochondria was visualized with the red fluorescent probe, Mitotracker CMXRos. **B, C**. Mitochondrial membrane depolarization in DU145 cells after treatment with 5 µM B28Bn(6–14). Detached cells were stained with JC-1 and subjected to FACS analysis (**B**). Adherent cells were dual stained with JC-1 and DAPI and observed under fluorescence and phase contrast (PCM) microscopes (**C**). The decrease of red fluorescence signal revealed the loss of mitochondrial transmembrane potential. Scale bar is 10 µm for all images.

Because the mitochondria-disrupting peptide, B28, exerts its activity at the mitochondria, we used FITC-labeled B28Bn(6–14) and a red fluorescent Mitotracker to examine the localization of the peptide. After treatment with 5 µM B28Bn(6–14), the mitochondria of DU145 cells were visualized with 10 ng/ml Mitotracker Red. It was found that the fluorescent B28Bn(6–14) largely localized to the mitochondria(Figure 3A, upper panel). But the amount of B28Bn(2–7) is smaller than that of B28Bn(6–14) accumulated in mitochondria (Figure 3A, lower panel). Next, we detected mitochondrial injury by analyzing the mitochondrial transmembrane potential. The cationic fluorescent dye, JC-1, exhibits potential-dependent accumulation in the mitochondria [26]. After treatment with B28Bn(6–14) and staining with JC-1, FACS analysis revealed that the ratio of red to green fluorescence decreased from 98.72/1.25 to 63.86/36.12 at 10 min and to 34.98/65.02 at 30 min, indicating the loss of mitochondrial transmembrane potential (Figure 3B). Moreover, mitochondrial injury was confirmed by a time-course analysis of JC-1 accumulation in mitochondria under a fluorescence microscope. Figure 3C shows that the cells displayed rich, granule-like, healthy mitochondria before peptide treatment. However, the number of healthy mitochondria gradually decreased, accompanied by nuclear condensation and fragmentation after treatment with B28Bn(6–14) from 10 min to 240 min. These results suggest that B28Bn(6–14) largely localizes in the mitochondria and induces mitochondrial depolarization after cellular internalization of the peptide.

### B28Bn(6–14) Induces Caspase-dependent Apoptosis in Tumor Cells

**Figure 4:**
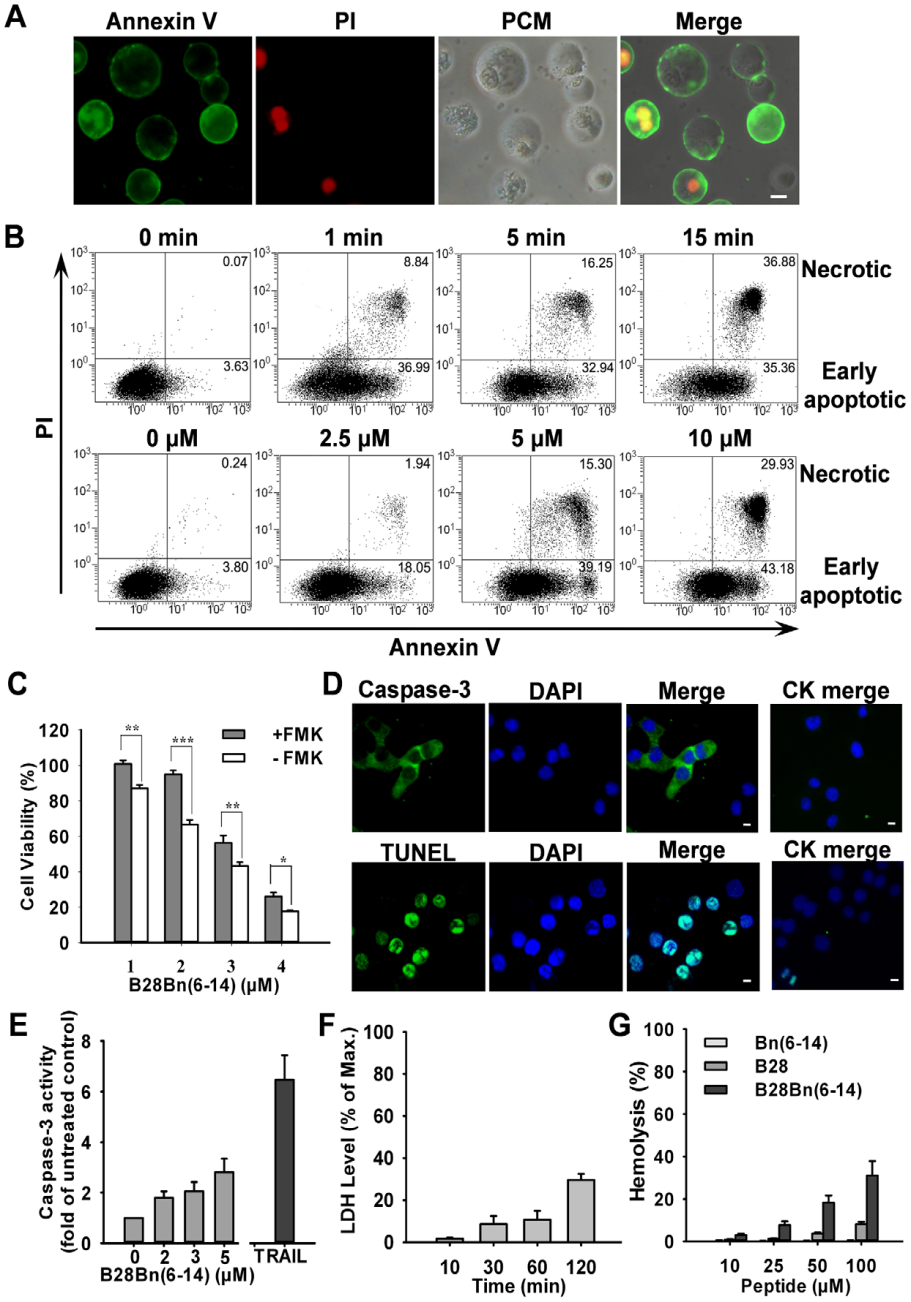
Effect of B28Bn(6–14)-induced apoptosis and necrosis in tumor cells. A . Detection of B28Bn(6–14)-induced apoptotic cells under a microscope. Cells treated with B28Bn(6–14) for 30 min were subjected to dual staining with FITC-Annexin V (green) and PI (red). Cells showing Annexin V−/PI-, Annexin V+/PI-, Annexin V+/PI+ were considered living, early apoptotic, and necrotic, respectively, in the merged image (PCM, phase contrast microscope). **B**. FACS analysis of B28Bn(6–14)-induced apoptosis. DU145 cells were either treated with 5 µM B28Bn(6–14) for different time, or treated with B28Bn(6–14) at different concentrations for 10 min. Cells were stained and analyzed by FACS. Cells in early apoptotic and necrotic stages are indicated as the percentage of total cells counted. **C**. Inhibition of B28Bn(6–14)-induced apoptosis with the Pan-caspase inhibitor, Z-VAD-FMK. Cells were pre-incubated with Z-VAD-FMK, treated with B28Bn(6–14), and cell viability was determined. *P<0.05, **P<0.01, ***P<0.001, Student’s *t* test. **D**. B28Bn(6–14)-induced caspase-3 activation and DNA fragmentation. Cells were treated with B28Bn(6–14) and stained either with the cleaved caspase-3 antibody or the TUNEL kit. Compared with untreated cells (CK merge), the positive cells presented green fluorescence with DAPI-stained nuclei. **E**. Caspase-3 Colorimetric Assay. Caspase-3 activity was presented as the fold of untreated cells. 2 µg/ml TRAIL was used as the positive control. **F**. Evaluation of B28Bn(6–14)-induced necrosis. After treatment with 5 µM B28Bn(6–14), the release of lactate dehydrogenase (LDH) into the culture supernatant was detected. LDH levels were presented as the percentage of the max release from the cells treated with 0.9% Triton X-100. **G**. Assessment of B28Bn(6–14)-induced hemolysis. Human erythrocytes in PBS were treated with the peptide for 16 h. Hemolysis was presented as the percentage of the absorbance at 540 nm from erythrocytes treated with 0.1% Triton X-100. Scale bar is 10 µM for all images.

When the cytoplasm is intact, exposure of phosphatidylserine (PS) on the outer leaflet of plasma membrane indicates cellular apoptosis. Annexin V binds to PS with high affinity. Cell-impermeable PI can only enter the cells when the plasma membrane is compromised. Therefore, Annexin V+/PI- cells were considered to be in the early stages of apoptosis [30]. After treatment with 5 µM B28Bn(6–14) and dual staining with Annexin V and PI, numerous DU145 cell were Annexin V+/PI- when visualized under a fluorescence microscope (Figure 4A). FACS analysis demonstrated that the percentage ratios of early apoptotic DU145 cells increased in a time- and peptide concentration-dependent manner. After treatment with 5 µM B28Bn(6–14) for 0, 1, 5, and 15 min, the percentages of early apoptotic DU145 cells were 3.63, 36.99, 32.94, and 35.36, respectively (Figure 4B, upper panel). Similarly, after treatment with 0, 2.5, 5, and 10 µM B28Bn(6–14) for 10 min, the percentage ratios of early apoptotic DU145 cells were 3.80, 18.05, 39.19, and 43.18, respectively (Figure 4B, lower panel).

To investigate the involvement of caspase in the observed peptide-induced apoptosis, the Pan-caspase inhibitor, Z-VAD-FMK, was preincubated with cells prior to addition of the peptide. Preincubation of DU145 with Z-VAD-FMK increased the cell viability, which ranged from 5% to 25% (Figure 4C). Moreover, caspase-3 activation was detected in the peptide-treated DU145 cells using immunofluorescence staining with an antibody against cleaved caspase-3 (Figure 4D, upper panel). Further fluorometric assays also verified the peptide-caused activation of caspase-3 in DU145 cells. As shown in Figure 4E, after treatment with 2–5 µM B28Bn(6–14), the activity of caspase-3 in peptide-treated DU145 cells is 2–3 times higher than that in untreated cells. In addition, TUNEL-positive cells were also observed in the peptide-treated DU145 cells (Figure 4D, lower panel). These results demonstrate that B28Bn(6–14) induces caspase-dependent apoptosis in DU145 cells.

Simultaneously, a few Annexin V+/PI+ cells were also observed in B28Bn(6–14)-treated DU145 cells under the fluorescence microscope (Figure 4A). FACS analysis also revealed a small population of Annexin V+/PI+ cells. After treatment with B28Bn(6–14) for 0, 1, 5, and 15 min, the percentages of Annexin V+/PI+ cells were 0.07, 8.84, 16.25, and 36.88, respectively (Figure 4B, upper panel). After treatment with 0, 2.5, 5, and 10 µM B28Bn(6–14) for 10 min, the percentage ratios of Annexin V+/PI+ cells were 0.24, 1.94, 15.30, and 29.93, respectively (Figure 4B, lower panel). Usually, the Annexin V+/PI+ cells were considered to be necrotic, irrespective of their origin [30]. A time-dependent release of LDH was also detected in B28Bn(6–14)-treated DU145 cells (Figure 4F). Although Annexin V+/PI+ cells might also be in the late stages of apoptosis, B28Bn(6–14)-induced necrosis in DU145 cells could not be excluded. To test the membrane-disrupting capability of the peptide, we incubated erythrocytes, which lack mitochondria [31], with B28Bn(6–14). As shown in Figure 4G, after incubation with 10 µM B28Bn(6–14) for 16 h, less than 5% lysis was detected in these peptide-treated erythrocytes. Moreover, only 5%-30% lysis was detected in erythrocytes incubated with B28Bn(6–14) at high concentrations, ranging from 25 to 100 µM. Simultaneously, the unconjugated Bn(6–14) and B28 induced less than 10% lysis of erythrocytes at 100 µM. These results suggest that B28Bn(6–14) mainly induces apoptosis in tumor cells, especially at low concentration, and the ability of B28Bn(6–14) to induce necrosis is limited.

### B28Bn(6–14) Suppresses Tumor Growth *in vivo*

**Figure 5:**
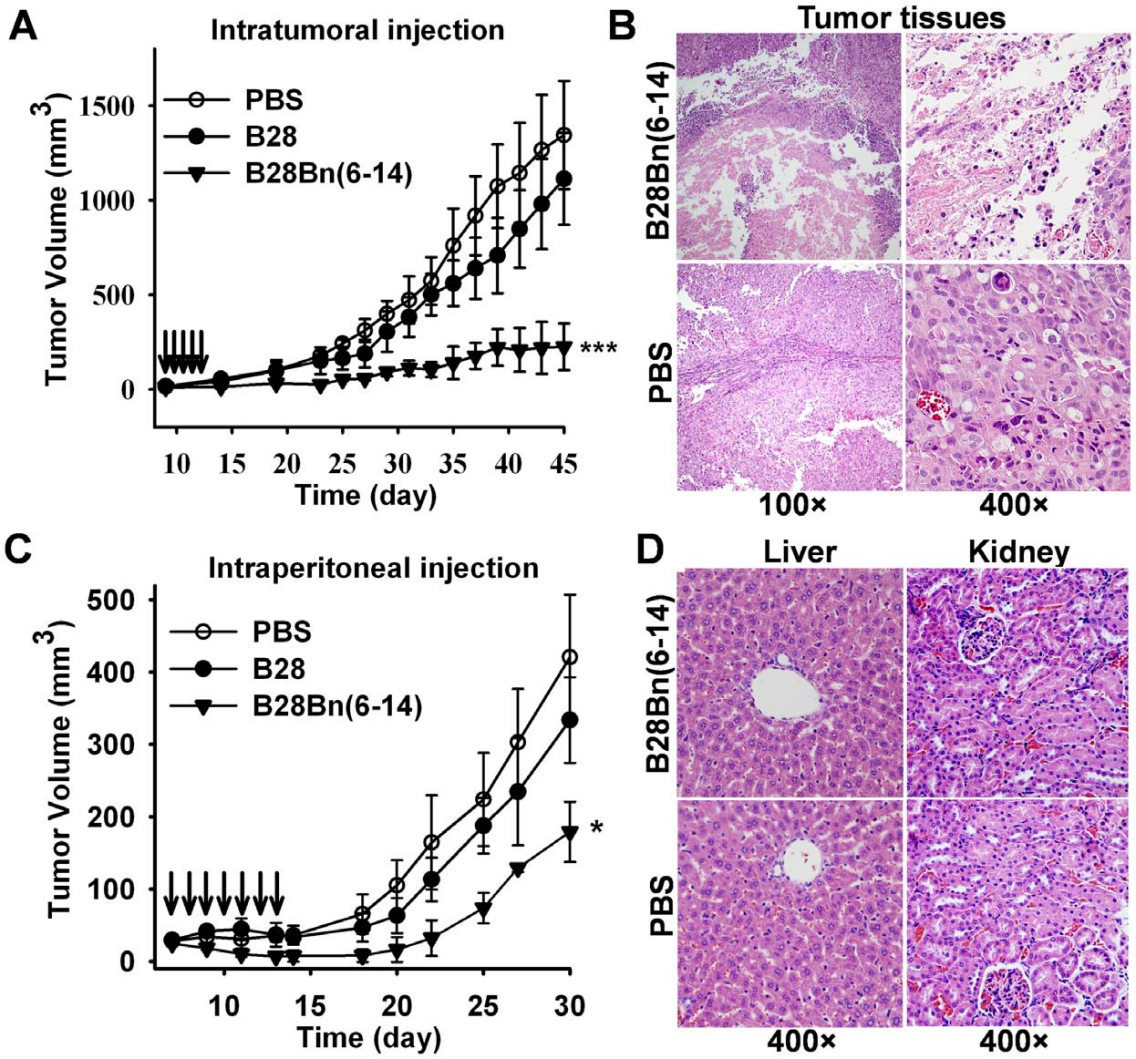
Effect of B28Bn(6–14) treatment on prostate tumor growth *in vivo*. A . Effect of intratumoral injection**.** BALB/c nude mice bearing DU145 tumors were administered the peptide (5 mg/kg) or PBS daily on days 9-14 (arrows-indicated) post inoculation. ***, *P*<0.001 versus PBS from day 23 to the end, or B28 from day 25 to the end. **B**. B28Bn(6–14)-induced tumor tissue disruption. Mice bearing DU145 tumor grafts (200–300 mm^3^) were intratumorally administered a single, 50 µl dose of 50 µg B28Bn(6–14) or PBS. At 24 h post injection, the animals were sacrificed and the tumor tissues were routinely stained with H&E. **C**. Effect of intraperitoneal injection**.** Mice bearing tumors received the peptide (15 mg/kg) or PBS on days 7-13 (arrows-indicated) post inoculation. *, *P*<0.05 versus PBS or B28 from day 13 to the end. **D**. *In vivo* cytotoxicity of the liver and kidney was determined by histological examination. At the end of i.p. therapy with B28Bn(6–14) or PBS, liver and kidney from the sacrificed animals were routinely stained with H&E. The histopathologic architecture was analyzed by light microscopy. The original magnification was indicated. The tumor volume was calculated as length×width^2^×0.5. Differences in tumor growth were analyzed by one-way ANOVA test.

To evaluate the efficacy of B28Bn(6–14) in tumor models, we established superficial DU145 prostate tumors in BALB/c nude mice. When the tumor volume reached 20-40 mm^3^, the mice were randomized into three groups (n = 5) and intratumorally injected with 5 mg/kg peptide or an equivalent volume of PBS, once daily for 5 consecutive days. As shown in Figure 5A, tumor growth in B28-treated mice was similar to that of PBS-treated mice. However, the intratumoral injection of B28Bn(6–14) substantially suppressed the growth of superficial tumors. From day 23 through completion of the study, the mean tumor volume in B28Bn(6–14)-treated mice was significantly different from that of the B28- (P = 0.009) and PBS-treated (P<0.001) groups. At the end (day 45) of the experiment, the mean tumor volumes of the PBS-, B28- and, B28Bn(6–14) -treated mice were 1344±286 mm^3^, 1113±243 mm^3^, and 225±124 mm^3^, respectively (Figure 5A). Histochemical analysis revealed that intratumoral injection of B28Bn(6–14) extensively disrupted tumor tissues at the injection site within 24 h (Figure 5B). The efficacy of intraperitoneal injection of B28Bn(6–14) was further evaluated in the DU145 xenograft model. At the onset of a palpable tumor (20-40 mm^3^), the mice (n = 5) received 15 mg/kg peptide or an equivalent volume of PBS, once daily for 7 consecutive days. As shown in Figure 5C, B28 was unable to suppress tumor growth. The tumor growth rate of B28-treated mice was similar to that of PBS-treated mice. However, intraperitoneal injection of B28Bn(6–14) exhibited an obvious tumor-suppressive effect. From day 13, the mean tumor volume in B28Bn(6–14)-treated mice was significantly different compared to the B28- (P = 0.034) and PBS-treated mice (P = 0.002). At the end of the experiment (day 30), the mean tumor volumes of the PBS-, B28-, and B28Bn(6–14)-treated mice were 421±87 mm^3^, 334±59 mm^3^, 179±42 mm^3^, respectively. In addition, there was no obvious histological damage in liver and kidneys of B28Bn(6–14)-treated mice (Figure 5D).

## Discussion

Bombesin and its analogues have been extensively used to selectively delivery small quantities of imaging and therapeutic agents into tumor tissues [8], [17], [18], [19]. Here, we report the ability of the peptide, Bn(6–14), which contains a bombesin receptor-binding motif, to deliver a mitochondria-disrupting peptide, B28. We found that Bn(6–14) selectively enhanced the cytotoxicity of B28 on tumor cells upon preferential binding of their conjugate, B28Bn(6–14), to tumor cells in a Bn(6–14) motif-dependent manner. Moreover, B28Bn(6–14) significantly suppressed the growth of superficial DU145 tumors after intratumoral or intraperitoneal injection *in vivo*. Our results suggest that Bn(6–14) effectively delivers the mitochondria-disrupting peptide to tumor cells.

The successful clinical use of somatostatin analogues such as ^111^In-penetreotide for tumor diagnosis and treatment has raised great interest in developing bombesin analogues via similar strategies [8], [32]. In contrast to the expression of somatostatin receptors, which are limited to neuroendocrine tumors, the substantial potential of bombesin as a drug vehicle largely benefits from the overexpression or ectopical expression of bombesin receptors, especially GRPR, in a wide range of lethal malignancies [8], [12], [33]. Bombesin-directed cytotoxic agents can selective kill tumor cells with cytotoxic cargos. These agents may also have the potential to interrupt the autocrine stimulatory effect as an antagonist due to the function of bombesin as an autocrine growth factor in many tumors [34], [35]. These characteristics of bombesin suggest the potential localization to and treatment of many common human cancers with bombesin-directed agents. It was reported that the C-terminal bombesin sequence, more than seven but no more than nine amino acids, is responsible for binding bombesin receptors [16]. To improve the affinity of the natural form of bombesin toward all receptors, especially GRPR, many bombesin analogues have been developed that focus on the modification and usage of the C-terminal bombesin residues. Among these agents, the BN-(6/7-14) analogues with the incorporation of unnatural amino acids are most frequently applied in preclinical and clinical studies [8], [18], [19]. Here, we selected the natural form of Bn(6–14) as a model of the peptide drug carrier because synthesis of the chimeric peptide is more convenient. Our results indicate these Bn(6–14) analogues have improved affinity for bombesin receptors and may also have the ability to carry a bioactive peptide.

Prostate and breast cancer cells, such as PC-3, DU145, and MCF-7, frequently overexpress bombesin receptors. These cells are usually used as models to identify the selectivity of various bombesin analogues *in vitro and in vivo* [8], [17], [18], [19]. To expose the active C terminus of Bn(6–14), we coupled B28 to the N terminus of Bn(6–14). The secondary structure prediction and determination suggested that both motifs in the conjugated B28Bn(6–14) showed homologous secondary structure compared with their unconjugated forms (Figure 1). As expected, Bn(6–14) significantly enhanced the cytotoxicity of B28 toward DU145 and PC-3 cells at 2–5 µM. Conversely, the B28-Bn(2–7) conjugate, B28Bn(2–7) lacking the C-terminal receptor-binding fragment of bombesin, exhibited much lower cytotoxicity than B28Bn(6–14) (Figure 2A). Lengthening the α helix in a cytotoxic peptide may enhance the ability to disrupt cell membrane and cytotoxicity [25], [36]. We found that B28Bn(2–7), which contains a longer predicted α helix presented slightly higher cytotoxicity than unconjugated B28 (Figure 2A). This problem can usually be attenuated by incorporating a linker between the leader and cytotoxic moiety. Further investigation found that 5 µM B28Bn(6–14) dramatically induced 60%-90% cell death in prostate and breast tumor cells. The IC_50_ of B28Bn(6–14) for tumor cells was 3–10 times lower than that of normal cells (Figure 2B). The selective internalization of B28Bn(6–14) in tumor cells was dependent on the Bn(6–14) motif and resulted in the selective cytotoxicity of B28Bn(6–14) (Figure 2D).

In cytotoxicity assays, we observed that B28Bn(6–14) induced drastic cell death within a short time (<60 min) (Figure 2). This rapid cell killing might be related to the fast internalization of B28Bn(6–14). It was reported that Both the Bn(6–14) analogue [13] and B28 (BMAP-28) [23] used in this experiment could be internalized into tumor cells within 10 min. We also observed that B28Bn(6–14) entered 80% DU145 cells within 30 min (Figure 2D). In addition, once enter cell, the peptide was accumulated in mitochondria (Figure 3A). The mitochondria-disrupting peptide, B28, is an inducer of the mitochondrial permeability transition pore [23]. We found that B28Bn(6–14) induced MMP and destroyed the mitochondrial membrane (Figure 3B, C), likely in a B28 motif-dependent manner. Once MMP happens, it leads to rapid, efficient cell death rapidly via multiple mechanisms, including apoptosis [20]. The early stages of apoptosis can be detected by the exposure of phosphatidylserine (PS) on the outer plasma membrane [30]. The PS assay showed that 5 µM B28Bn(6–14) induced the emergence of numerous early apoptotic (30%-40%) and necrotic (10%-40%) cells within 15 minutes (Figure 4B). Moreover, caspase activation including caspase-3 and the signs of late apoptotic DNA fragmentation were detected after B28Bn(6–14) treatment (Figure 4C-E). These results indicate that B28Bn(6–14) predominantly induced apoptosis especially after treatment with at low concentration of peptide for a short time. Flow cytometer analysis demonstrated that the ratio of necrotic cell (Annexin V+/PI+) to early apoptotic cell (Annexin V+/PI-) increased along the prolongation of treating-time and increase of peptide concentration (Figure 4B). However, the number of necrotic cells detected by LDH assay was less than 10% after B28Bn(6–14) treatment for 1 h (Figure 4F). In addition, erythrocytes lack cellular organelles, including the mitochondria, and the function to undergo mitochondrial apoptosis [31]. The resistance of erythrocytes to B28Bn(6–14)-treatment (around 5% lysis) below 25 µM confirmed that B28Bn(6–14) was unable to disrupt the plasma membrane and induce cell necrosis at low concentrations. The lysis ratios of erythrocytes gradually increased from 5%–30% when the peptide concentrations reached 25–100 µM (Figure 4G). These results suggest that necrosis might become the major death mechanism of cell treated with high concentration of B28Bn(6–14) for a long time.

The mitochondrial membrane of bacterial origin and is largely negatively charged [37]. The mammalian plasma membrane usually consists of neutral phospholipids and cholesterol, even though cancer cell membranes carry partial net negatively charged molecules. Therefore, the mammalian plasma membrane is relatively resistant to disruption by cationic antimicrobial peptides, including B28 and its chimeric peptides [23], [36]. In addition, the invalidation of the apoptotic response in cancer has been reported in experimental and clinical studies due to a variety of strategies, particularly the inactivation of MMP to limit or circumvent apoptosis in tumor cells [20], [38]. Strategies, including those used in this study, that disrupt the mitochondrial membrane may bypass this mechanism and overcome drug resistance [20], [22], [39].

Finally, we evaluated the anti-cancer efficacy of B28Bn(6–14) *in vivo*. Intratumoral and intraperitoneal administration of B28Bn(6–14) significantly suppressed the growth of prostate tumors in a mouse model without any obvious side effects (Figure 5). On the other hand, the suppressive effect on tumor growth was decreased with intraperitoneal administration compared to intratumoral injection. One crucial reason for this might be the short half-life of the peptide, which is comprised of only the natural form of amino acids *in vivo*. This indicates that strategies such as PEGylation and the incorporation of unnatural amino acids into the peptide should be considered to prolong the half-life of B28Bn(6–14) *in vivo* [11], [40]. Although many cancer biomarkers have been applied in developing anti-cancer drugs, less progress has been made in the development of clinical tools to predict the applicability of these targets in patients [41]. Bombesin receptors are not always overexpressed in individual tumor patients [8], [12], [33]. Further development of radiolabeled bombesin analogues that already exist for tumor imaging in humans may be helpful to select for patients who will be suitable for clinical trials and benefit from bombesin-directed therapeutic agents, such as B28Bn(6–14).

In conclusion, we constructed the chimeric peptide, B28Bn(6–14), for the purpose of evaluating the ability to specifically deliver a cytotoxic peptide, B28, to tumor cells by using Bn(6–14) as the vehicle. Our results suggest that Bn(6–14), containing the receptor-binding residues of bombesin, can substantially enhance the selective cytotoxicity of this mitochondria-disrupting peptide to bombesin receptor-overexpressing tumor cells and its anti-cancer efficacy *in vivo* without any obvious systemic toxicity. The Bn(6–14)-directed B28Bn(6–14) has been shown to be internalized, localize at mitochondria, and induce tumor cell apoptosis by efficiently causing mitochondrial depolarization. Thus, this smaller and non-exclusive bombesin analogue may provide opportunities to deliver diverse cytotoxic peptides specifically to tumor cells, enhancing targeted anti-cancer therapy in the future.
